# Recent progress in DNA marker-assisted selection for vegetable breeding in Japan

**DOI:** 10.1270/jsbbs.25049

**Published:** 2026-02-20

**Authors:** Tsukasa Nunome, Mitsuyo Kawasaki, Tomohiro Kakizaki, Koji Miyatake, Mitsuhiro Sugiyama, Yosuke Yoshioka, Satoshi Fujito, Masanori Honjo

**Affiliations:** 1 Institute of Vegetable and Floriculture Science (NIVFS), National Agriculture and Food Research Organization, 3-1-1 Kannondai, Tsukuba, Ibaraki 305-8519, Japan; 2 Institute of Vegetable and Floriculture Science (NIVFS), National Agriculture and Food Research Organization, 360 Ano, Tsu, Mie 514-2392, Japan; 3 Institute of Life and Environmental Sciences, University of Tsukuba, Tsukuba, Ibaraki 305-8572, Japan; 4 Tohoku Agricultural Research Center, National Agriculture and Food Research Organization, 4 Akahira, Shimokuriyagawa, Morioka, Iwate 020-0198, Japan

**Keywords:** marker-assisted selection, vegetable, breeding

## Abstract

The development of new cultivars exhibiting high levels of disease resistance and superior quality is critical for vegetable production. Recent advances in genomics and DNA sequencing technologies have facilitated the identification of sequence variants associated with specific phenotypes. This has enabled the development of novel and improved DNA markers for integration into marker-assisted selection (MAS). The application of MAS has significantly enhanced the efficiency of stacking loci responsible for disease resistance and quality characteristics. This paper provides an overview of recent research findings related to DNA markers and their applications in breeding in Japan, focusing primarily on vegetables from *Brassicaceae*, *Solanaceae*, *Cucurbitaceae*, and *Allium*, as well as strawberries.

## Introduction

Vegetables are essential sources of vitamins, minerals, fibers, and other nutrients that maintain a healthy diet. These plants exhibit considerable diversity in biological characteristics, such as reproductive systems (autogamy or allogamy, with self-incompatibility in some cases), generation length, ploidy level, and genome size. Consequently, the development of comprehensive reference genomes and DNA markers has progressed unevenly across vegetable species.

In Japan, the total value of agricultural production was 9.4953 trillion yen, of which vegetables accounted for 2.3243 trillion yen (24.5%) (Ministry of Agriculture, Forestry and Fisheries (2023), https://www.maff.go.jp/j/tokei/kouhyou/nougyou_sansyutu/index.html). The production values of major vegetables were as follows: tomatoes (231.1 billion yen), strawberries (205.5 billion yen), bunching onions (150.3 billion yen), cucumbers (141.3 billion yen), onions (127.3 billion yen), cabbages (100.2 billion yen), eggplants (82.5 billion yen), spinach (78.8 billion yen), lettuce (77.8 billion yen), daikon radishes (77.4 billion yen).

The total demand for vegetables has declined by about 10% over the past two decades. At the same time, demand has gradually shifted from household consumption to processing and food service use, which now accounts for approximately 60% of total vegetable demand. In response to this trend, vegetables are increasingly expected to meet requirements for year-round stable supply and consistent quality.

Vegetable production in Japan is influenced by several distinctive agricultural conditions that present current challenges requiring consideration. These include intensive cultivation on limited farmland, continuous cropping practices, and a rapidly aging farming population. In addition, vegetable production in Japan is expected to respond to the growing demand for processed products and increasing consumer interest in health-promoting traits. These factors have influenced breeding priorities.

Climate change, particularly rising temperatures, has introduced uncertainty in vegetable production conditions. Developing cultivars with resistance to disease and pests, tolerance to heat stress, and adaptability to fluctuating conditions is essential for sustainable production. Bolting in cabbage, radish, and onion is an important breeding trait because it is caused by specific temperature and day-length conditions, resulting in reduced product quality. The demand for a year-round supply of strawberries has expanded breeding efforts, including the development of everbearing cultivars.

Consumer preferences favor vegetables such as tomatoes, melons, and strawberries with superior taste attributes, such as high sugar content and excellent flavor. Growing interest in health has introduced nutritional and functional traits as a new breeding objective. This has prompted the development of cultivars that can accumulate specific beneficial components.

In fruit-bearing vegetables, fruit set occurs through insect pollination or the application of fruit-setting agents. Temperature extremes can reduce insect activity and lead to poor fruit set. Additionally, there is an increasing demand to reduce the labor required for applying fruit-setting agents. To address these issues, parthenocarpic cultivars that set fruit without pollination are being developed for eggplants, tomatoes, sweet peppers, and melons.

In Japan, the development and supply of vegetable cultivars are primarily led by private seed companies, although public research institutions and universities also contribute to the breeding of selected vegetables. Most vegetables currently distributed in the market are F_1_ hybrids, reflecting the emphasis on stable quality and yield. As a result, detailed information on commercially developed cultivars is often not publicly disclosed. In this article, we summarize DNA markers commonly used in major vegetable crops (Table 1), review leading cultivars developed through genetic analysis and MAS in *Brassicaceae*, *Solanaceae*, *Cucurbitaceae*, *Allium* species, and strawberries, examine the current status and challenges of vegetable breeding in Japan.

## Brassicaceae vegetables

*Brassicaceae* includes widely cultivated vegetables like cabbage, Chinese cabbage, and radish. Most *Brassicaceae* vegetables require vernalization for bud differentiation, and some require vegetative growth for several weeks to acquire vernalization competence. Therefore, the generation time is relatively long. MAS can eliminate undesirable plants early and is indispensable for *Brassicaceae* breeding. We developed a clubroot resistant cultivar using DNA marker selection. Clubroot is a soil-borne disease causing significant damage across Japan and is difficult to control. Additionally, we developed DNA markers and cultivars for late bolting in Chinese cabbage, and secondary metabolites in Japanese radish.

Clubroot is a severe soil-borne disease of *Brassicaceae* caused by the protozoan *Plasmodiophora brassicae* Woronin. Resting spores in the soil survive long term and are difficult to control through crop rotation, agrochemicals, or other agricultural practices. Resistant cultivars are considered most effective for controlling clubroot. Among the more than 20 clubroot resistance (CR) genes or loci identified in *Brassica rapa* (Hasan *et al.* 2021), *Crr1*, *Crr2* and *CRb* are mainly used for breeding in Japan. The two loci derived from the European turnip ‘Siloga’, *Crr1* and *Crr2*, confer resistance to pathotype groups 1, 2, and 4, especially when combined (Hatakeyama *et al.* 2004, Suwabe *et al.* 2003). *Crr1* is composed of a major gene *Crr1a* (encoding a TIR-NB-LRR protein) and a gene *Crr1b* with minor effects (Hatakeyama *et al.* 2013, Suwabe *et al.* 2012). *CRb*, which is identical to *CRa*, confers resistance to pathotype groups 3 (Hatakeyama *et al.* 2017, Kato *et al.* 2012, 2013, Piao *et al.* 2004, Ueno *et al.* 2012). Chinese cabbage F_1_ cultivar ‘Akimeki’ was developed using marker-assisted backcrossing. ‘Akimeki’ harbors *Crr1*, *Crr2*, and *CRb*, confers resistance to all four pathotype groups. *B. rapa* has also been used as CR donor in the breeding of amphidiploid *Brassica* crops. CR rapeseed cultivar ‘CR nanashikibu’ harboring *Crr1* and *Crr2* was developed using marker-assisted backcrossing with the resynthesized *Brassica napus* by interspecific crossing between CR Chinese cabbage line ‘PL9’ and a cabbage cultivar (Kawasaki *et al.* 2021). The application of MAS has enabled the efficient and precise transfer of CR genes, thereby accelerating the breeding of not only Chinese cabbage cultivars but also amphidiploid rapeseed cultivars.

For *Brassicaceae* vegetables, bolting time is a critical breeding trait, particularly for cultivars harvested from spring to early summer. Premature bolting in an inappropriate season severely affects harvest quality. The bolting pathway in *Brassicaceae* plants has been well characterized in *Arabidopsis* (Niu *et al.* 2024). *FLOWERING LOCUS C* (*FLC*), encodes a MADS-box protein, represses bolting in a dose-dependent manner by binding to the regulatory elements of the floral inducer genes *FT* and *SOC1* (Helliwell *et al.* 2006, Michaels and Amasino 1999). This pathway is conserved across *Brassicaceae* species, and *FLC* function loss results in early flowering (Su *et al.* 2018). Expression of *FLC* is suppressed by prolonged low temperatures. Continuous *FLC* expression that does not respond to low temperatures is known to delay bolting. Large insertions in the first introns of *BrFLC2* and *BrFLC3* in Chinese cabbage (*B. rapa* L. ssp. *pekinensis*), homologs of *Arabidopsis* *FLC*, prevent *FLC* suppression and maintain bolting inhibition (Kitamoto *et al.* 2014). DNA markers detecting this insertion are used in late bolting breeding programs. The Chinese cabbage F_1_ cultivar ‘ITOSAI No. 1’, selected for late bolting *BrFLC2* and *BrFLC3* alleles, demonstrates the most robust late bolting among commercial cultivars (Kitamoto *et al.* 2023). In spring cultivation, heating has traditionally been used to prevent bolting caused by exposure to low temperatures. However, ‘ITOSAI No. 1’ exhibits a high vernalization requirement for bolting, allowing cultivation without the need for heating.

Glucosinolates (GSLs) are secondary metabolites in *Brassicaceae* vegetables that contribute to odor and pigmentation of harvested and processed products (Ishida *et al.* 2014). Glucoraphasatin, the primary GSL in radishes (*Raphanus sativus* L.), is responsible for the distinct smell and yellow color of Takuan-zuke (Takahashi *et al.* 2015). The GSL composition of radish has been uniform, with cultivars containing high glucoraphasatin levels. However, screening of radish genetic resources revealed a mutant where glucoerucin was the dominant GSL (Ishida *et al.* 2015). Genetic analysis revealed that this strain carries a mutation in *GLUCORAPHASATIN SYNTHASE 1* (*GRS1*), the enzyme that converts glucoerucin into glucoraphasatin (Kakizaki *et al.* 2017). Radish plants homozygous for the defective *GRS1* allele exhibit minimal glucoraphasatin accumulation. Using this defective allele of *GRS1*, it is possible to modify the GSL biosynthetic pathway in radishes, enabling breeding of cultivars lacking characteristic odors and pigments. Developing a DNA marker to detect *GRS1* mutations will enable the rapid breeding of radish cultivars without glucoraphasatin production, eliminating the need for GSL analysis. Using these DNA markers, several F_1_ cultivars without glucoraphasatin have been developed since 2015. One cultivar, ‘Sarah White’, has high dry matter content in roots, making it suitable for processed ingredients like grated daikon radish (daikon-oroshi) and dried strips (kiriboshi-daikon) (Ishida and Morimitsu 2020). Given its non-discoloring and odorless properties, development of glucoraphasatin-free cultivars for various processing applications is expected to continue.

## Solanaceae vegetables

*Solanaceae* includes important vegetables like tomatoes (*Solanum lycopersicum* L.), eggplants (*Solanum melongena* L.), and sweet peppers (*Capsicum annuum* L.). In Japan, vegetables grown in open fields during spring/summer and are forced from autumn to summer, enabling year-round supply. *Solanaceae* vegetables have been bred in Japan for a long time with private seed companies and public institutions have developed many excellent cultivars.

As listed in Table 1, tomatoes face various pathogens, including viruses, fungi, and bacteria, and resistance genes have been studied using DNA markers. According to private seed company catalogs, these characteristics are actively introduced. Resistance to *Tomato yellow leaf curl virus* (TYLCV) is the most significant viral challenge. *TYLCV resistance gene 1* (*Ty-1*) is widely used for TYLCV-resistant breeding in Japan, and resistant cultivars have been developed by Japanese seed companies. Koeda and Kitawaki (2024) used DNA marker analysis to reveal the introduced genes. Although MAS usage for cultivars was not explicitly stated by private seed companies, multiple companies developing multiple resistant cultivars with the same genes suggests that DNA markers were used.

Although *Tomato mosaic virus* (ToMV) resistance is an essential trait, *ToMV resistance gene 2^2^* (*Tm-2^2^*) shows stable resistance in various races, which can be addressed using the markers currently in use (Koeda and Kitawaki 2024, Verlaan *et al.* 2013).

Root-knot nematodes resistance is another important trait. The root-knot nematode resistance gene *Mi-1.2* is widely used in most tomato cultivars (Furumizu and Sawa 2023). Nematodes bypassing this resistance gene are emerging as threats (Marques de Carvalho *et al.* 2015).

Bacterial diseases pose a major threat to *Solanaceae* vegetable production. Among them, bacterial wilt, caused by *Ralstonia solanacearum*, is one of the most serious diseases. The most well-known resistance quantitative trait loci (QTLs) are bacterial wilt resistance on chromosomes 6 (*Bwr-6*) and 12 (*Bwr-12*) (Genin and Denny 2012). Even when these two QTLs are introduced, the resistance of current rootstock cultivars falls short of the desired level, suggesting multiple genes are involved and identifying causative genes is challenging. Attempts to introduce resistance genes into rootstock cultivars have resulted in cultivars with various levels of resistance to bacterial wilt.

There are examples of selection markers for resistance genes in sweet peppers. The most common application of MAS is breeding for resistance to tobamoviruses, including *Pepper mild mottle virus* (PMMoV). Four resistance genes, *L^1^*, *L^2^*, *L^3^*, and *L^4^*, have been identified, conferring resistance to pathotypes P_0_, P_1_, P_1,2_, and P_1,2,3_, respectively (Matsunaga *et al.* 2003). Recently developed cultivars have specified resistance gene types, suggesting marker utilization.

This provides examples of disease resistance-selective markers in *Solanaceae* vegetables. We have introduced examples of viral resistance. However, when breeding *Solanaceae* vegetables using viral resistance genes, an important issue must be considered. In *Solanaceae* vegetables, grafting onto rootstocks is common to confer resistance against soil-borne diseases. When using rootstocks, if scion and rootstock have different resistance genotypes, the graft interface may exhibit hypersensitivity reactions owing to the resistance response to viruses, leading to plant death. Therefore, identifying resistance gene types using markers is important in breeding, not only for conferring resistance but also for avoiding graft incompatibility. This eliminates labor-intensive inoculation of multiple standard viruses, improving breeding efficiency.

Another important trait is the conferral of labor-saving properties. We introduce parthenocarpy, extensively studied in tomato and eggplant. These characteristics freed farmers from regular fruit-setting agent treatment. Since the causative genes, *parthenocarpic fruit 2* (*pat-2*) and *k* (*pat-k*), have been isolated and markers published (Nunome *et al.* 2013, Takisawa *et al.* 2018), their use is expected to increase to alleviate labor shortages. In eggplant, the effect of parthenocapy is more pronounced because fruits develop singly rather than in clusters like tomatoes. Many cultivars developed in Japan recently exhibit parthenocarpy. The first research on parthenocarpy in Japan used the parthenocarpic eggplant cultivar ‘Talina’ from Europe. With DNA marker development advancement in eggplant, research progressed rapidly, leading to two highly effective QTLs, controlling parthenocarpy 3.1 (*Cop3.1*) and 8.1 (*Cop8.1*) (Miyatake *et al.* 2012), which are utilized in the breeding parthenocarpic cultivars, such as ‘YAMAGATA N 1GO’, developed by the National Agriculture and Food Research Organization (NARO) and Yamagata Prefecture. The parthenocarpic gene, *parental advice-1*, found in cultivar, ‘PC Chikuyo’ (Takii & Co., Ltd.), exhibits highly stable parthenocarpy as a single factor and has been introduced into commercial cultivars, leading to cultivar replacement in major production areas. This gene has been isolated (Matsuo *et al.* 2020) and can be introduced into any genetic background.

The absence of prickles on calyxes, stems, and leaves, is a desirable trait in eggplant, contributing to improved handling efficiency and can be selected using a DNA marker (Miyatake *et al.* 2020). The development of new prickleless eggplant cultivars has been reported, and the use of this DNA marker is expected to facilitate further breeding.

## Cucurbitaceae vegetables

*Cucurbitaceae* vegetables, including cucumber (*Cucumis sativus* L.), melon (*Cucumis melo* L.), squash (*Cucurbita* spp.), and watermelon (*Citrullus lanatus* (Thunb.) Matsum. et Nakai), are cultivated in open fields from spring to autumn, and in greenhouses throughout the year in Japan. Diseases have caused major problems, reducing yield and fruit quality. Genetically conferred host resistance is ideal for overcoming disease control problems worldwide. The development of resistant cultivars is a primary objective of modern breeding programs. DNA markers are widely used for disease resistance breeding in Japanese *Cucurbitaceae* vegetables. Traits for high fruit quality and labor-saving cultivation are important, and genetic analysis and DNA marker development have been conducted in Japan.

The whole-genome sequence of cucumbers was first reported in 2009 (Huang *et al.* 2009), and various cucumber lines have been resequenced. A high-grade genome reference of ‘Tokiwa’, a Japanese old cultivar, was reported in 2025 (Seiko *et al.* 2025). Numerous genetic studies have identified resistance genes and loci in various wild and cultivated cucumbers (Wang *et al.* 2020).

Major fungal and oomycete diseases infecting cucumber cause significant yield losses in Japan. These include powdery mildew (PM), mainly caused by *Podosphaera* *xanthii*; downy mildew (DM), caused by *Pseudoperonospora cubensis*; target leaf spot (TLS) caused by *Corynespora cassiicola*; and anthracnose (AR), caused by *Colletotrichum orbiculare*. Cucumber is susceptible to soil-borne fungal diseases like Fusarium wilt (FW), caused by *Fusarium oxysporum* f. sp. *melonis*. However, cucumbers are commonly grafted onto squash in Japan; soil-borne diseases resistance is not prioritized. Angular leaf spot (ALS), caused by *Pseudomonas syringae*, is a bacterial disease that is a major problem in the cucumber-producing areas of Japan. Additionally, various viral diseases caused by *Cucumber mosaic virus* (CMV), *Zucchini yellow mosaic virus* (ZYMV), *Melon yellow spot virus* (MYSV), and *Cucurbit chlorotic yellows virus* (CCYV) have increased in recent years.

Genetic mapping studies for resistance to PM, DM, AR, ZYMV, and MYSV have been conducted in Japan, identifying several resistance loci (Amano *et al.* 2013, Fitriyah *et al.* 2024, Fukino *et al.* 2013, Sugiyama *et al.* 2015, Yoshioka *et al.* 2014). Genetic analyses of fruit shape and texture traits (firmness and crispness) have been conducted (Shimomura *et al.* 2017, 2021), and DNA polymorphisms near QTLs could be used as DNA markers in breeding.

Many major diseases in melons are similar to those in cucumbers with some differences. Unlike cucumbers, melons in Japan are almost unaffected by TLS and relatively unaffected by DM and AR. FW resistance is crucial for developing melon rootstock cultivars. The whole-genome sequence of melon was reported in 2012 (Garcia-Mas *et al.* 2012), and a high quality genome reference of ‘Earl’s Favorite Harukei 3’, was reported in 2020 (Yano *et al.* 2020). This genomic information has facilitated genetic mapping and DNA marker development. Similar to cucumber, MAS has been commonly adopted in melon breeding. The following are important DNA markers and examples of disease-resistant cultivars recently developed using DNA markers in Japan.

The melon cultivars of ‘Earl’s Aporon’ series with CCYV resistance were developed through MAS using simple sequence repeat (SSR) markers in 2024 (Kawazu *et al.* 2024). CCYV was first reported in Japan and is transmitted by the sweet potato whitefly, *Bemisia tabaci* (Gyoutoku *et al.* 2009). I-10 (JP 138332) has been reported resistant to CCYV (Okuda *et al.* 2013) and was crossed with ‘Earl’s Favorite Harukei No. 3’ to develop the ‘Melon Chukanbohon Nou 5 Go’, with CCYV resistance (Sugiyama *et al.* 2019). QTL analysis using I-10 identified one locus on chromosome 1, and SSR markers linked to the resistance gene were developed (Kawazu *et al.* 2018). ‘Melon Chukanbohon Nou 5 Go’ was crossed with Earl’s Favorite type cultivars, and backcrossing introduced the resistance genes using MAS and CCYV resistance tests. The CCYV resistance to I-10 is recessive; therefore, phenotypic identification is impossible in individuals heterozygous for resistance genes. MAS has been effectively used in backcross breeding to develop these cultivars.

The genes *Fom-1* and *Fom-2* confer resistance to FW caused by races 0 and 1, and 0 and 2, respectively (Oumouloud *et al.* 2012, 2015). The *nsv* gene confers resistance to MYSV (Morales *et al.* 2005, Nieto *et al.* 2006), and *PMQU2.1* and *PMQU12.1* confer resistance to PM caused by *P. xanthii* pxA and pxB strains (Diaz *et al.* 2015, Fukino *et al.* 2008). DNA markers that identify fruit flesh color (the gene *CmOr*) have been used (Tzuri *et al.* 2015). Genetic analyses of parthenocarpy and pistil-bearing flowers on the main stem have been conducted (Nashiki *et al.* 2023, https://doi.org/10.1101/2023.02.16.528896). DNA polymorphisms near these QTLs have been used or could be used as DNA markers in melon breeding in Japan.

Among *Cucurbitaceae* vegetables, squash (especially *C. maxima*) and watermelon are also important in Japan. Genomic information for these crops has accumulated, and genetic analyses of disease resistance have progressed. Major diseases of squash include PM, gummy stem blight, phytophthora rot, and mosaic diseases, such as CMV, ZYMV, and *Watermelon* *mosaic* *virus*. Disease resistance studies have focused on *C. moschata* and *C. pepo*, but not *C. maxima*, which is the most important species in Japan. The major PM resistance locus (*Pm*-*0*) in several *C. moschata* and *C. pepo* cultivars were introduced from a wild relative by interspecific hybridization long ago. The location of *Pm*-*0* and candidate genes have been reported for each species (Sabharwal *et al.* 2024). A Japanese seed company patented a DNA marker for PM resistance in *C. maxima* and developed a new PM-resistant cultivar. The source of the resistance gene is unclear; however, resistance may be conferred by the above or a homologous gene. Although several DNA markers exists for *C. pepo* and *C. moschata*, a few reports cover *C. maxima*.

Common diseases in Japanese watermelon fields include AR, PM, and CCYV. Genetic analyses were conducted for AR and PM resistance. Race differentiation exists for AR (races 1 and 2 appear are most common). A non-synonymous single nucleotide polymorphism (SNP) in *Cla001017*, encoding a coiled-coil nucleotide binding site leucine-rich repeat (CC-NBS-LRR) protein, confers strong resistance to race 1 (Jang *et al.* 2019). This mutation is conserved among cultivars resistant to race 1 (Matsuo *et al.* 2022), including the commercial cultivars in Japan. No promising source was found in race 2.

Most improvements in *Cucurbitaceae* vegetables rely on conventional breeding methods. The superiority of the current cultivars is a testament to the success of these methods. However, there are several problems with this approach, such as being time-consuming and less precise, particularly when aiming to improve complex traits governed by multiple QTLs. Genetic analyses of useful traits of *Cucurbitaceae* vegetables have been conducted in Japan to address this issue. Several effective sources of resistance against major diseases have been discovered, and effective DNA markers for resistance have been developed (Table 1). Further discovery of useful gene sources and establishment of a MAS system should progress in the future, leading to the development of new cultivars with multiple disease resistance and other favorable agronomic traits.

## Allium vegetables

Conventional breeding of *Allium* vegetables takes many years because these are annual or biennial. Therefore, the development of practical DNA markers can reduce the effort required for breeding. However, genome research has made little progress until approximately 2020 because of their large genome size (Ricroch *et al.* 2005), the outcrossing of plants, and their proneness to inbreeding depression. Thus, most developments have been conducted using conventional selection rather than DNA marker selection.

Even in such a situation, analyses have been conducted to develop DNA markers linked to the traits of vegetables of the *Allium* genus using bulb onions and shallots, where it is possible to produce genetically fixed doubled haploids and a complete set of *Allium fistulosum* L.- *Allium. cepa* monosomic addition lines (MALs) (Abdelrahman *et al.* 2015, Shigyo *et al.* 1996) as materials. MALs are strains in which one chromosome from shallots has been added to the Japanese bunching onion, and the expression of traits from each shallot chromosome makes it possible to perform genetic analysis of anthocyanin and flavonoid production in the leaf sheath (Shigyo *et al.* 1997) and rust resistance traits (Wako *et al.* 2015). A high-density linkage map was constructed from the expressed gene information for each chromosome obtained using these MALs and the F_2_ population between the doubled haploid bulb onion and doubled haploid shallot. Information on these expressed genes has been compiled in AlliumTDB (https://alliumtdb.kazusa.or.jp/) (Fujito *et al.* 2021), and later effectively utilized in the analysis of the whole genome sequence of bulb onions and the development of chromosomal markers for useful traits. A research group centered at Wageningen University and Research succeeded in linking the genome information of doubled haploid bulb onions into a pseudomolecule for each chromosome, and contributed to verifying the validity of the sequence using this information as an anchor marker (Finkers *et al.* 2021).

Additionally, a DNA marker set was developed in bulb onions to efficiently perform linkage analysis of the entire chromosome (Sekine *et al.* 2022). It is possible to develop DNA markers linked to desired traits efficiently by utilizing this DNA marker set. At the time, there was no chromosome-level reference genome assembly; therefore, Sekine *et al.* (2022) comprehensively examined the sequence information of expressed genes in the leaves of two cultivars that differed in traits such as bulb size and identified expressed genes whose sequences differed between the cultivars. Next, by utilizing the information on expressed genes for each chromosome mentioned above, they selected expressed genes such that they were uniformly distributed across the entire chromosome and developed DNA markers that could be used to analyze DNA polymorphisms between cultivars.

Bulb onion genomic information has been used to create DNA markers and linkage maps for Japanese bunching onions. In Japanese bunching onions, markers linked to multiple traits have been developed through QTL analysis, reducing the labor required for selecting traits that are time-consuming and labor-intensive to investigate by using markers. DNA markers that identify low pungency have been developed for Japanese bunching onions (Tsukazaki *et al.* 2012), because it is difficult to directly quantify sulfur-containing compounds, which are the pungent components of Japanese bunching onions, and conduct sensory tests at a large scale. The markers can only be used with some breeding materials, and that low-pungency QTLs are homozygous in the parental lines of F_1_ cultivars. The cultivars distinguishable by these markers are limited to the specific cultivars used in each test; therefore, it is desirable that marker development becomes available for a greater number of cultivars in the future.

DNA markers have been developed to assess the bolting time of Japanese bunching onions (Wako *et al.* 2016). In Japan, bunching onion flower bolts from spring to early summer and late-bolting cultivars are in high demand to prevent the loss of commercial value. When the main QTL for late bolting is homozygous, the bolting date is delayed compared to when it is heterozygous or when it does not have QTL for late bolting.

The number of cultivars developed using these markers for breeding *Allium* vegetables is limited. It is important to provide markers linked to more important traits and develop markers that can be used for a wide range of breeding materials in a simple and easy-to-use manner to increase the efficiency of MAS. In recent years, the development of long-read sequencing technologies has enabled the construction of high-accuracy genomes at a low cost, even for crops with large genome sizes, leading to the release of reference genomes for three species of *Allium* plants (Liao *et al.* 2022). The development of DNA markers is expected to accelerate as it has become easier to develop them using genomic information.

## Strawberry

More than 90% of strawberry fruits are produced from winter to spring by forcing culture of June-bearing cultivars. However, because strawberries are in demand throughout the year, everbearing cultivars, which can bear fruit in summer and autumn when June-bearing cultivars do not bear fruit under natural conditions, are used for fruit production. Because cultivation continues for a long period of time, pest control is an important issue. Therefore, important breeding targets for strawberries include flowering characteristics, such as early/late flowering and everbearing, which affect the harvest period, disease resistance, yield characteristics, and fruit traits, such as flavor, color, firmness, and shape.

The use of DNA markers is expected to improve strawberry breeding efficiency. However, cultivated strawberries possess a highly heterozygous octoploid genome (2n = 8x = 56, Bringhurst 1990, Edger *et al.* 2019, Kunihisa 2011), and this complex genome structure has hindered the construction of comprehensive reference genome sequences and the development of DNA markers linked to agronomically significant traits.

DNA markers have been developed in Japan for agriculturally important qualitative traits, such as the everbearing flowering trait (Honjo *et al.* 2016, 2020, Saiga *et al.* 2023), anthracnose resistance (Enoki *et al.* 2014, Iimura *et al.* 2012), Fusarium wilt resistance (Iimura *et al.* 2021) and PM race 0 resistance (Koishihara *et al.* 2016), where the presence of major genes is suggested by phenotypic segregation patterns. Various breeding programs in Japan are starting to use these markers, and the development of excellent breeding materials is progressing (Saido *et al.* 2024).

Recent advances in genome sequencing technology and analysis protocols, such as long-read sequencing techniques, have yielded comprehensive reference genome sequences and high-quality haplotype-phased genomes of octoploid strawberries. (Edger *et al.* 2019, Han *et al.* 2025, Hirakawa *et al.* 2014, Isobe *et al.* 2020, Jin *et al.* 2025, https://doi.org/10.1101/2021.11.03.467115). In addition to these precedents, the reference sequence for one of the major cultivars in Japan, i.e., ‘Koiminori’, is scheduled to be released soon. Moreover, a genetic analysis method for ploidy QTLs applicable to octoploid strawberries has been established, which is expected to facilitate the genetic analysis of significant traits (Yamakawa *et al.* 2021). Yamakawa *et al.* (2025) identified three QTLs associated with red fruit flesh color based on the above-mentioned procedure; by selecting individuals that retained all three DNA markers, a high selection frequency of 92% for red-fleshed individuals was achieved. Red flesh color is important for processed products such as jams, purees, and cakes. Yamakawa *et al.* (2025) identified an early maturity QTL and a de-maturity QTL that alleviated the early maturity effects. By selecting individuals that retained the DNA marker for the former QTL, but lacked the marker for the latter QTL, a selection frequency of 90% was attained for early maturing individuals. Early maturing cultivars are in high demand for forcing culture because they can produce fruit on time to meet the demand for strawberries for Christmas cakes.

Currently, strawberry cultivars developed using MAS are not commercially available in Japan. Although cultivars with diverse flowering traits and disease resistance have been developed, they have been selected based on phenotypic evaluation. The development of DNA markers is expected to accelerate breeding efforts, including the selection of cultivars with improved flowering characteristics and disease resistance.

## Future perspective

Exploration of genetic resources possessing novel and beneficial traits is essential for future breeding efforts, and the development of unique resources is actively underway. However, developing these populations requires the long-term cultivation of large populations and trait evaluation. A core collection is a representative subset of accessions selected from a vast collection of genetic resources to cover genetic diversity efficiently. In Japan, core collections have been established for vegetables such as eggplants, cucumbers, and melons (Miyatake *et al.* 2019, Shigita *et al.* 2023, 2024). The eggplant core collection, accompanied by genomic data, has been characterized for practical agricultural traits, including disease resistance. Since these accessions have been confirmed for local culinary use, the introduction of useful traits can proceed without significant barriers. A four-way cross-population derived from the F_2_ population of two commercial F_1_ tomato cultivars has been developed (Ohyama *et al.* 2017). This population originated from four parental lines of the original F_1_ tomato cultivars and exhibited higher genetic diversity than the population derived from a cross between two parental lines. Multi-parent advanced generation intercross (MAGIC) populations are created by intercrossing multiple parental lines to promote extensive genome-wide recombination. This process yields a population that combines high genetic diversity with a minimal population structure and a broad spectrum of recombinant haplotypes. MAGIC populations have been established in strawberries, demonstrating significant value for both genetic analysis and breeding applications (Tasaki *et al.* 2020, Wada *et al.* 2017).

In Japan, DNA markers and whole-genome sequence data for vegetables have been curated and are widely accessible through databases such as Plant Garden (https://plantgarden.jp/ja/index), Melonet-DB (https://melonet-db.dna.affrc.go.jp/), and AlliumTDB (https://alliumtdb.kazusa.or.jp/). These resources facilitate genetic analyses based on genome-wide polymorphism data, thereby accelerating the identification of novel genes for valuable traits using core collections. Genome-wide association study (GWAS) have been conducted on vegetables such as tomatoes (Yamamoto *et al.* 2024) and strawberries (Wada *et al.* 2020). Moreover, genomic prediction, which estimates phenotypic performance from genome-wide polymorphism data, has enabled genomic selection for breeding purposes. Genomic selection has been implemented in vegetables such as tomatoes (Yamamoto *et al.* 2017) and strawberries (Nagano *et al.* 2018), contributing to improved selection accuracy for complex traits, including yield and fruit quality. These advanced genomic approaches are expected to significantly enhance breeding efficiency and precision of trait selection.

Recent advances in genome-related technologies have significantly expanded vegetable breeding possibilities. Progress in long-read sequencing technologies has enabled the establishment of high-quality reference genome assemblies, including haplotype phasing. This advances the analysis of how genomic structural variation influences trait expression. Furthermore, automation of phenotyping data collection is expected to enhance the speed and precision of breeding processes. The integration of these technologies is expected to accelerate the identification of valuable genetic resources and the establishment of MAS systems, leading to the more efficient development of new cultivars.

## Author Contribution Statement

MK and TK wrote the draft for the section on *Brassicaceae* vegetables; KM for the section on *Solanaceae* vegetables; MS and YY for the section on *Cucurbitaceae* vegetables; SF for the section on *Allium* vegetables; MH and TN for the section on strawberries; and TN integrated all the sections of the text. All the authors have reviewed and approved the final manuscript.

## Figures and Tables

**Table 1.** DNA markers used in marker-assisted breeding in vegetables

Species	Trait	Locus/gene	Chromosome/Linkage group	References
*Brassica rapa*	Clubroot resistance	*Crr2*	A01	Suwabe *et al.* 2003
		*CRb*	A03	Piao *et al.* 2004, Kato *et al.* 2012, 2013
		*Crr1a*	A08	Suwabe *et al.* 2003, Hatakeyama *et al.* 2013
		*Crr1b*	A08	Hatakeyama *et al.* 2013
	Late bolting	*BrFLC2*, *BrFLC3*	A02, A03	Kitamoto *et al.* 2014, Kitamoto *et al.* 2017
*Raphanus sativus*	Glucosinolate composition	*GRS1*	R01	Ishida *et al.* 2015, Kakizaki *et al.* 2017
*Solanum lycopersicum*	ToMV resistance	*Tm-1*	Chr. 2	Ohmori *et al.* 1995
		*Tm-2/Tm-2^2^*	Chr. 9	Shi *et al.* 2011, Panthee *et al.* 2013
	TYLCV resistance	*Ty-1/Ty-3/Ty-3a*	Chr. 6	Verlaan *et al.* 2013, Koeda and Kitawaki 2024
		*Ty-2*	Chr. 6	Yamaguchi *et al.* 2018, Kim *et al.* 2020
	TSWV resistance	*Sw-5*	Chr. 9	Tong *et al.* 2023
	Fusarium wilt resistance	*I-1*	Chr. 11	Sarfatti *et al.* 1991, Catanzariti *et al.* 2017
		*I-2*	Chr. 11	Simons *et al.* 1998
		*I-3*	Chr. 7	Catanzariti *et al.* 2015
	Fusarium crown and root rot resistance	*Frl*	Chr. 9	Devran *et al.* 2018
	Leaf mold resistance	*Cf-4/Cf-9*	Chr. 1	Truong *et al.* 2011, Thomas *et al.* 1997
		*Cf-2/Cf-5*	Chr. 6	Dickinson *et al.* 1993, Dixon *et al.* 1998
	Verticillium wilt resistance	*Ve1*	Chr. 9	Kawchuk *et al.* 2001
	Corky root rot resistance	*py-1*	Chr. 3	Doganlar *et al.* 1998
	Gray leaf spot resistance	*Sm*	Chr. 11	Yang *et al.* 2022
	Powdery mildew resistance	*Ol-1*	Chr. 6	Bai *et al.* 2005, Lian *et al.* 2022
	Late blight resistance	*Ph-3*	Chr. 9	Zhang *et al.* 2013
	Bacterial wilt resistance	*Bwr-6*, *Bwr-12*	Chr. 6, Chr. 12	Kim *et al.* 2018
	Bacterial canker resistance	*Rcm6*	Chr. 6	Abebe *et al.* 2022
	Root-knot nematode resistance	*Mi-1.2*	Chr. 6	Furumizu and Sawa 2023
	Parthenocarpy	*pat-2*	Chr. 4	Nunome *et al.* 2013
		*pat-k*	Chr. 1	Takisawa *et al.* 2017
*Solanum melongena*	Fusarium wilt resistance	*Fm1*	Chr. 2	Miyatake *et al.* 2016
	Parthenocarpy	*Cop3.1*, *Cop8.1*	Chr. 3, Chr. 8	Miyatake *et al.* 2012
		*pad-1*	Chr. 3	Matsuo *et al.* 2020
	Prickleless	*pl*	Chr. 6	Miyatake *et al.* 2020, Li *et al.* 2024
		*PE*	Chr. 6	Zhang *et al.* 2024
*Capsicum annuum*	PMMoV resistance	*L^3^*	Chr. 11	Sugita *et al.* 2004
		*L^4^*	Chr. 11	Matsunaga *et al.* 2003
	Root-knot nematode resistance	*N*	Chr. 9	Wang *et al.* 2009
		*Me1*	Chr. 9	Wang *et al.* 2018a
		*Me3/Me7*	Chr. 9	Liu *et al.* 2023
	TSWV resistance	*Tsw*	Chr. 10	Jahn *et al.* 2000, Moury *et al.* 2000
	Phytophthora blight resistance	*phyt-1*, *phyt-2*, *phyt-3*	LG7, LG1, LG15	Sugita *et al.* 2006
			LG15, LG3	Minamiyama *et al.* 2007
		*CaPhyto*	Chr. 5	Wang *et al.* 2016
		*Pc5.1*	Chr. 5	Bongiorno *et al.* 2023
*Cucumis sativus*	downy mildew, anthracnose, angular leaf spot resistance	*CsSGR*	Chr. 5	Wang *et al.* 2018b
	powdery mildew resistance	*CsMLO1*, *CsMLO8*, *CsMLO11*	Chr. 1, Chr. 5, Chr. 6	Berg *et al.* 2017, Dong *et al.* 2024
	target leaf spot resistance	*cca-3*	Chr. 6	Wen *et al.* 2015
	ZYMV resistance	*VsVPS4*	Chr. 6	Amano *et al.* 2013
	anthracnose resistance	*An5*	Chr. 5	Fitriyah *et al.* 2024
	MYSV resistance	*Sws*	Chr. 3	Sugiyama *et al.* 2015
*Cucumis melo*	CCYV resistance		Chr. 1	Kawazu *et al.* 2018
	Fusarium wilt resistance	*Fom-1*, *Fom-2*	Chr. 11	Oumouloud *et al.* 2012, 2015
	MNSV resistance	*nsv*	Chr. 12	Morales *et al.* 2005, Nieto *et al.* 2006
	powdery mildew resistance	*PMQU2.1*, *PMQU12.1*	Chr. 2, Chr. 12	Fukino *et al.* 2008, Diaz *et al.* 2015
	flesh color	*CmOr*	Chr. 9	Tzuri *et al.* 2015
*Cucurbita* spp.	powdery mildew resistance	*Pm-0*	Chr. 3	Sabharwal *et al.* 2024
*Citrullus lanatus*	anthracnose resistance	*Cla001017*	Chr. 8	Jang *et al.* 2019, Matsuo *et al.* 2022
	powdery mildew resistance	*ClaPMR2*	Chr. 2	Mandal *et al.* 2020
*Allium cepa*	CMS	*coxI*, *orf725*	mtDNA	Kim *et al.* 2009
*Allium fistulosum*	low pungency		2A	Tsukazaki *et al.* 2012
	bolting time		1A, 2A	Wako *et al.* 2016
*Fragaria* × *ananassa*	flowering habit			Honjo *et al.* 2016, 2020, Saiga *et al.* 2023
	anthracnose resistance			Enoki *et al.* 2014, Iimura *et al.* 2012
	Fusarium wilt resistance			Iimura *et al.* 2021
	powdery mildew resistance			Koishihara *et al.* 2016
	fruit flesh color			Yamakawa *et al.* 2025
	fruit maturity			Yamakawa *et al.* 2025
